# Radiative nanobubbles in aqueous solutions: Rayleigh instability sustains resonant oscillations and potential long-range interactions

**DOI:** 10.3389/fchem.2026.1885538

**Published:** 2026-08-21

**Authors:** Nikolai F. Bunkin, Yulia V. Novakovskaya, Sergey A. Tarasov, Vladimir S. Boriskin, Evgenii V. Zubkov, Angelina A. Boriskina, Alexey V. Smirnov, Polina N. Borisoglebskaya, Olga V. Fartushnaya, Anastasia O. Petrova, Natalia N. Rodionova, German O. Stepanov

**Affiliations:** 1 Department of Fundamental Sciences, Bauman Moscow State Technical University, Moscow, Russia; 2 Chair of Physical Chemistry, Chemistry Department, Lomonosov Moscow State University, Moscow, Russia; 3 Research and Development Department, OOO “NPF “Materia Medica Holding”, Moscow, Russia; 4 Laboratory of Physiologically Active Substances, Institute of General Pathology and Pathophysiology, Moscow, Russia; 5 Department of General and Medical Biophysics, Institute of Biomedicine, Pirogov Russian National Research Medical University, Ministry of Health of the Russian Federation, Moscow, Russia

**Keywords:** dynamic light scattering, electromagnetic radiation, gas nanobubbles, gigahertz range, hydrated electrons, Rayleigh instability, thermography, vibrational treatment

## Abstract

**Introduction:**

Vibrational treatment increases gas nano- and microbubble content in aqueous environments. Nanobubbles stabilized by ions (bubstons) generate electromagnetic waves in the GHz range during regular oscillations and can reversibly capture secondary electrons by their boundary hydration shells. We hypothesize that these emissions facilitate long-range interactions between aqueous solutions separated by glass walls.

**Methods:**

To test this hypothesis, we investigated the long-range coupling between an inner NaCl solution (10 mg/L) contained in a glass vial and various outer immersion liquids, including untreated water, vibrationally treated water, and NaCl solutions. The effects of mechanical shaking and atmospheric exposure on liquid dynamics were evaluated using highresolution thermography and theoretical modeling.

**Results:**

We demonstrate that while mechanical shaking promotes nanobubble formation, evaporation creates internal temperature gradients that trigger Rayleigh instability and turbulent convective flows in atmospheric-exposed outer samples. Rayleigh instability in liquids induces and supports oscillations of nanobubbles, which cause wave emission. Thermographic and theoretical analysis confirms that these intense convective flows drive the oscillations and cause charge redistributions within the bubbles’ hydration shells.

**Disscusion:**

Our findings provide a possible mechanism showing how Rayleigh instability supports resonant nanobubble oscillations, suggesting that convective flows are a driver of long-range interactions in aqueous solutions.

## Introduction

For many substances, including biological macromolecules, experimentally detectable radiation is emitted from their aqueous solutions. When the radiation is generated due to a chemical reaction, the phenomenon is referred to as chemiluminescence; for biological systems, it is known as bioluminescence (Harvey and Newton, 1957; Lee, 2017; Naumova et al., 2018; Vacher et al., 2018; Vladimirov and Proskurnina, 2009). Notably, the electromagnetic radiation from solutions of biological macromolecules spans frequencies from radio waves to the near-ultraviolet range (Cifra et al., 2011).

Recent studies describe long-range interactions between biological systems, where the physical nature of the radiation is not related to chemiluminescence. For example, it was shown that vibrational treatment (shaking) of two closely spaced vials, where one vial is filled with a solution of biological macromolecules and the other is filled with ultra-pure water, can cause changes in the properties of the latter (e.g., THz spectra, GHz radiation, etc.) (Don et al., 2025; Petrova et al., 2024). These changes are also observed as a result of multiple, consecutive vibrational treatments. However, it remains unclear to what extent the physical mechanism responsible for these effects produced by an aqueous solution of macromolecules on ultra-pure water is related to radiative processes, since the efficiency of the effect depends on the duration of vibrational treatment (Petrova et al., 2024). A relatively recent study has also shown that dilute aqueous solutions of bacterial and viral DNAs emit low-frequency electromagnetic radiation (Montagnier et al., 2015). Additional studies report that long-term radiofrequency irradiation inactivates bacteria (Espitia et al., 2025; Yakymenko et al., 2016), enhances chemiluminescence in protein solutions; and modifies physicochemical properties of aqueous solutions (Astashev et al., 2023; Gudkov et al., 2020). Vibrationally treated solutions were also reported to produce long-range effects on other solutions, increasing their cell-activation capacity by over 7-fold (Novikov and Yablokova, 2022). This long-range influence depends on the external weak magnetic fields and disappears when the field is shielded (e.g., at field intensities of ∼10–20 nT) (Novikov et al., 2024; Novikov and Yablokova, 2022). Thus, radiative processes often accompany vibrational effects that enable long-range influence between aqueous solutions.

It is well known that, despite the diversity of mechanical effects, their characteristics can be reduced to a few key parameters, namely, frequency, acceleration, force, and duration (Gudkov et al., 2025). The magnitude of the mechanical effects varies by the type of system.

From a physical point of view, vibrational treatment of water and aqueous solutions should cause substantial distortions and rupture of the hydrogen bond network, promoting the dissolution of atmospheric gases and the formation of nanobubbles (Bunkin et al., 2025; Novakovskaya et al., 2025). The mechanical stability of nanobubbles and their stability against the outward diffusion of gases require the compensation of the surface tension, which is achieved through the localization of ions in the water layers surrounding the gas core of the nanobubble (Bunkin et al., 2025; Nirmalkar et al., 2018). This concept aligns with the experimental findings showing that gas nanobubble lifetimes increase with an increasing concentration of an electrolyte added to water (Yakovlev et al., 2024).

NaCl is a key electrolyte in biological systems (Chudy et al., 2025). The chloride anion in NaCl is a typical chaotropic ion, which can efficiently be included in the boundary layers of gas nanobubbles in water, localizing directly at the gas-liquid interface (Novakovskaya et al., 2025). In the absence of specially added ions in water, which is in equilibrium with the atmosphere and, hence, contains CO_2_, bicarbonate anions can be built in the boundary hydration layers (which are at least three molecules thick) surrounding the gas cores of nanobubbles, providing stabilization of the nanobubble (Bunkin et al., 2025). Note that in ultra-pure water long-exposed to atmospheric air and saturated with CO_2_, the hydrolysis of the latter (H_2_O + CO_2_ ↔ H^+^ + HCO_3_^−^) predetermines the pH value of 5.5, which corresponds to a bulk concentration of ions of ∼ 10^17^ cm^−3^ (Yan et al., 2018).

Nanobubble stabilization is crucial for the following reasons (Wang et al., 2023): In a nanobubble with a radius *R*_*b*_ ≈ 100 nm, the inner gas pressure far exceeds the outer pressure at the liquid-gas interface (as follows from the Laplace equation and quantum chemical simulations (Bunkin et al., 2025)), which should cause the gas outleakage into the bulk liquid and the subsequent nanobubble collapse (Epstein and Plesset, 1950). Diffusion-unstable nanobubbles have lifetimes proportional to *R*_*b*_^2^ (Ljunggren and Jan Christer, 1997), lasting just 0.1 ms at *R*_*b*_ = 100 nm. If ions of the same sign (anions or cations) in the liquid can be localized within the surface layers of a nanobubble, a charged spherical shell around the gas core is formed, and an electrostatic component of the Helmholtz free energy is equal to $\Phi_{е}\left(r\right)=\frac{1}{2\varepsilon }\int_{R_{b}}^{r}\left[Q_{0}^{2}\left(x\right)/x^{2}\right]\;dx$ , where *Q*_0_ is the charge of the nanobubble boundary layer, (*r-R_b_*) is its thickness, ε = 82 is the permittivity of water (this and subsequent equations are written in the CGSE system).

The specific free energy related to the electrostatic component results in the appearance of a ponderomotive pressure $P_{e}=-{\left(\frac{\partial \Phi_{е}}{\partial V}\right)}_{T}$ (where T is the temperature), which stretches a spherical bubble. Here, $V=\frac{4\pi }{3}\left(r^{3}-R_{b}^{3}\right)$ is the volume of the space external to the bubble.

In this case, the Laplace equation can be rewritten as

Pin+Pe=P0+2ΓRb (1)

where *P*_*in*_ is the gas pressure inside the bubble, *P*_0_ is the atmospheric pressure, and Г is the surface tension coefficient.

Under certain conditions, the 2Г⁄*R*_*b*_ = *P*_*e*_ equality in Equation 1 is met, which means that the stretching ponderomotive pressure counterbalances the surface tension forces compressing the bubble. Such a bubble will acquire both mechanical (equality of inner and outer pressures) and diffusion stability (inner gas pressure equals the atmospheric pressure). We named such bubbles “bubstons”, which is an abbreviation for “bubble stabilized by ions” (Bunkin and Bunkin, 2016; Bunkin et al., 2016; Yurchenko et al., 2016).

Bubstons undergo radial and ellipsoidal oscillations (the latter being volume-preserving deformations). Analysis of theoretical models has shown that both types of oscillations are characterized by frequencies of 1–3 GHz (Bunkin et al., 2025). These frequencies fall within the range, in which the emission of radiation from water and aqueous NaCl solutions increases after vibrational treatment. This study explores the possible role of the discovered dynamical peculiarities of nanobubbles in producing long-range effects.

In our previous studies, we observed that vibrationally treated solutions, which contain nanobubbles, demonstrate an increased absorbance at 600 nm upon the high-energy electron beam irradiation. This indicates an increase in the number of hydrated (secondary) electrons (Novakovskaya et al., 2025). At the same time, the formation/disappearance kinetics of secondary electrons also changes due to their efficient (reversible in the radiation field) localization in the hydration boundary layers of gas nanobubbles. Thus, all other conditions being the same and in the absence of any other outer forces, a change in the absorbance at 600 nm (upon the irradiation with a high-energy electron beam) may indicate a change in the bulk content of nanobubbles in the solution (Novakovskaya et al., 2025).

In our previous study, vibrational treatment of liquid samples was accomplished through mechanical shaking with varying intensities (Bunkin et al., 2025). It is known, however, that any liquid exposed to the atmosphere undergoes surface evaporation, meaning the temperature at the liquid surface will be lower than the bulk temperature. This process creates a temperature gradient within the liquid layer, and under certain conditions, Rayleigh instability occurs, which results in turbulent hydrodynamic flows in the liquid. The primary motivation for our study was to clarify whether charged nanobubbles can emit electromagnetic waves in a liquid sample exposed to the atmosphere due to Rayleigh instability, and whether this radiation can alter the properties of a wall-separated liquid sample poured in a tightly sealed vessel immersed in the open outer sample. Long-term exposure of the sealed liquid sample to a sample open to the atmosphere is referred to as incubation hereafter. During the incubation process, we cannot change the intensity of mixing that occurs due to the Rayleigh instability, unlike our previous experiments, where we could vary the intensity of mechanical shaking of liquid samples. Therefore, the only control parameter that changes the sample’s characteristics in the sealed vial, is the duration of the incubation process. Notably, some of the samples used for incubation were initially (prior to incubation) subjected to intense vibrational treatment.

## Materials and methods

### Preparation of solutions and incubation process

NaCl was purchased from Sigma Aldrich, St. Louis, MO, USA. Ultrapure water type 1 with a specific resistance of 18.2 MΩ × cm at 25 °C obtained using a Milli-Q system (Millipore, Merck KGaA, Darmstadt, Germany) was used for the preparation of 10 mg/L NaCl solution and all other samples.

First, the samples were equilibrated with the atmosphere for one hour. Then, one group of samples underwent vibrational treatment solely, while the second group was subjected to vibrational treatment combined with a series of at least twelve successive centesimal (100-fold) dilutions. This process resulted in a theoretical concentration reduction of at least 10^24^. Manual shaking was performed at a frequency of approximately 4 Hz (21 blows in ∼4.8 s) with a force of 4 N (kg × m/s^2^). The frequency and intensity of shaking were monitored with a custom-made Dynamizer device using a Tenzometry Unit online monitoring software ver.3.1.1 developed based on the Laboratory Virtual Instrumentation Engineering Workbench (LabVIEW 2019, Version 1.4.15.5.). Samples were prepared in 40 mL vials (Glastechnik Grafenroda, Germany), poured to 500 mL sealed glass flasks (Simax, Czech Republic), and stored in the dark at room temperature.

**FIGURE 1:**
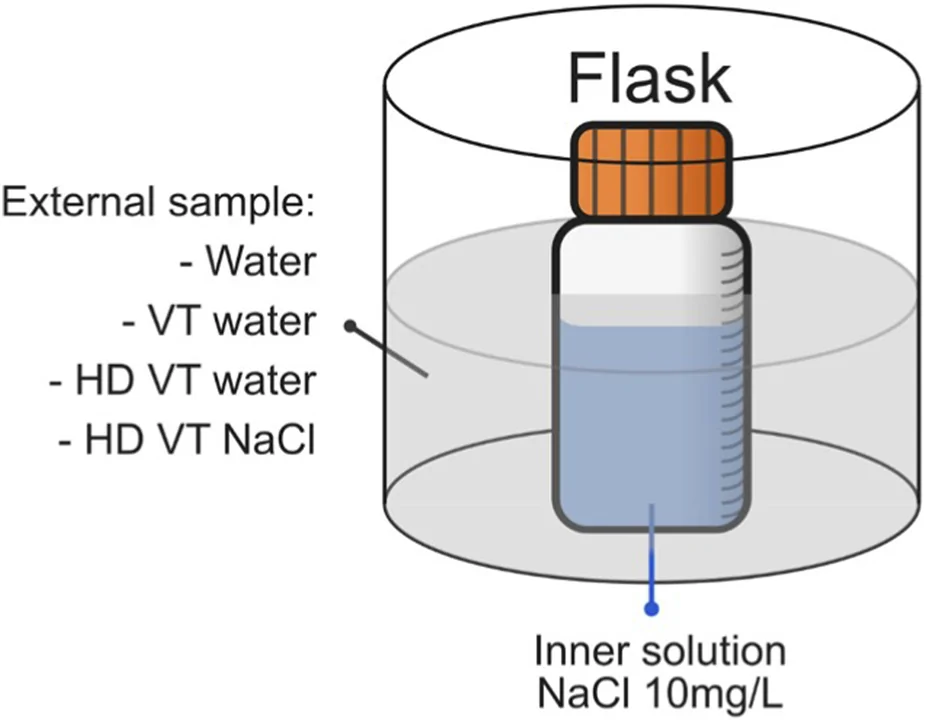
A 500-mL flask with an inner sample (NaCl solution, 10 mg/L) is immersed in a 2-L container filled with an outer sample. Incubation lasts for up to 4 h depending on the particular experiment. Outer solution: untreated water (Water), vibrationally treated water (VT water), highly diluted vibrationally treated water (HD VT water), and highly diluted vibrationally treated NaCl solution (HD VT NaCl).

For the incubation, a 500 mL flask filled with 10 mg/L NaCl solution (inner sample) was immersed in an external vessel filled with 2 L of the outer sample. The incubation duration varied from 0 to 4 h depending on a particular experimental protocol (see the scheme in Figure 1). The outer samples were as follows.

1. “Water”, reference water (no vibrational treatment);
2. “VT water”, vibrationally treated water (which was shaken vigorously);
3. “HD VT water”, highly diluted and vibrationally treated water;
4. “HD VT NaCl”, highly diluted and vibrationally treated NaCl (which was shaken vigorously during the repeated dilution procedures).

### Quantitative photometric analysis of radiation-absorbing particles

**FIGURE 2:**
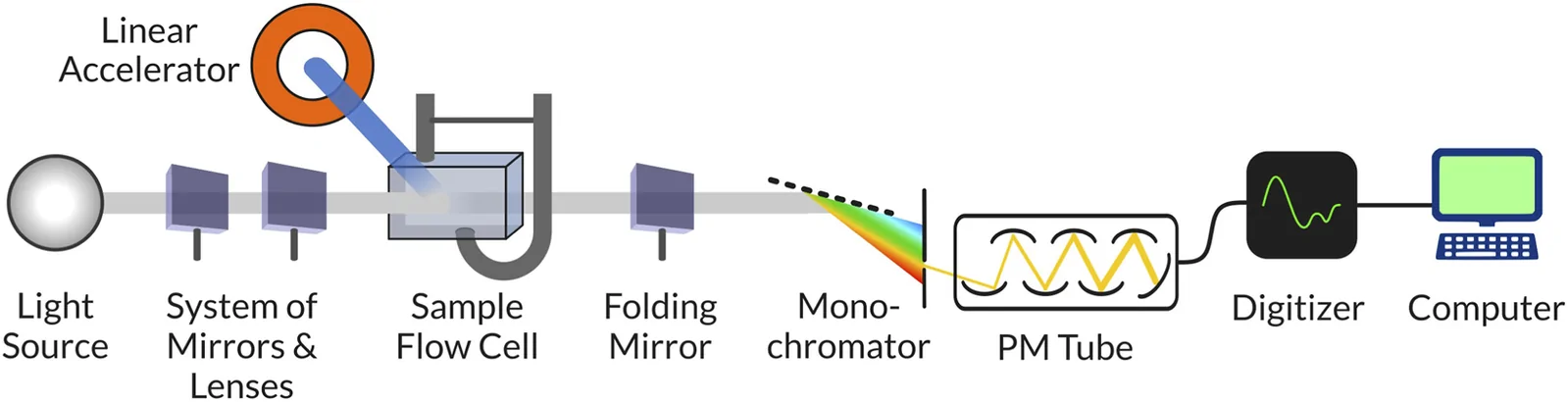
A scheme of the experimental setup for absorbance measurements at *λ* = 600 nm immediately after the high-energy electron beam impact.

The experimental setup is shown in Figure 2. A 100-mL flow-through cuvette (2) filled with the liquid sample was irradiated with an electron beam from a linear accelerator (1) using the same experimental installation and parameters as those described in (Novakovskaya et al., 2025).

A linear electron accelerator generated electron pulses of 1.5-µs duration with a current of 150 mA, which corresponds to approximately N = 1.5 × 10^12^ electrons with an energy of 6 MeV. The cuvette provided an optical path length of 10 mm along the beam axis. Simultaneously, the sample was illuminated with a broadband white light source (a 150 W xenon lamp with a spectral range of 250–800 nm). Immediately upon the electron-beam impact, the absorbance at *λ* = 600 nm was recorded as a function of time.

**FIGURE 3:**
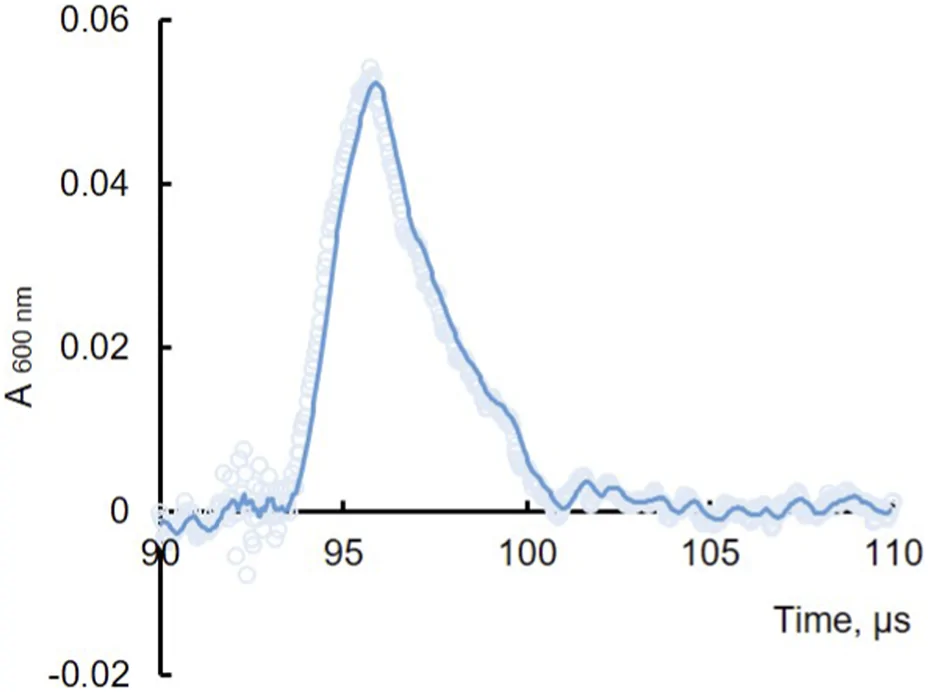
Temporal absorbance profile of hydrated (secondary) electrons at *λ* = 600 nm. The onset of the signal increase coincides with the high-energy electron impact.

Figure 3 illustrates the characteristic absorbance kinetics of hydrated electrons at *λ* = 600 nm. As previously established, the temporal profile of the signal is sensitive to the presence of ions (sodium and chloride ions) and nanobubbles in the system. In particular, the peak value increases, while the decay rate of the signal noticeably decreases with an increase in the content of nanobubbles (Novakovskaya et al., 2025).

### DLS determination of nanobubble contents

The size distribution and bulk content of nanobubbles were determined by DLS using a Zetasizer Ultra/Pro (Malvern, UK). The corresponding technique is described in detail in (Berne and Pecora, 2000; Chu, 1974). The DLS setup is equipped with a continuous He−Ne laser (λ = 633 nm, maximum power 4 mW) and a built-in temperature controller. The scattering angle was 173°.

### Laser phase microscopy

The Laser Phase Microscopy (LPM) method allows one to determine the phase shift profiles δ between the reference and object waves, which give an interference pattern on the pixels of the receiving matrix after the object wave passes through a particle in a liquid sample, see our recent work (Bunkin et al., 2021) for more details. The quantity δ is measured in units of λ/2, where λ is the wavelength. If the object wave passes through a spherical particle with diameter *d*, transparent to radiation, then the average value of the optical path difference (OPD) Δ*h*, measured at the maximum of the interference pattern, is determined by the formula $\Delta h=\frac{\delta \cdot \lambda }{2\pi \alpha }=\frac{d\cdot \Delta n}{\alpha }$ . Here α is a hardware coefficient, which depends on the particle size. In the approximation of geometric optics, we have α = 2. Thus, by measuring Δ*h*, it is possible to distinguish suspended particles with a higher or lower refractive index compared to the surrounding liquid, meaning that we can distinguish a gas bubble from a solid particle. In these experiments, we used a laser phase modulation interference microscope MIM-310 (Amphora Labs, Russia) operating at a wavelength λ = 405 nm.

### Microwave radiometric analysis of aqueous environments

Radiometry experiments were carried out using a TES-92 electromagnetic field detector (TES Electrical Electronic Corp., Taiwan). The device features a tri-axis (isotropic) sensor with a measurement range of 1 μW/m^2^ to 30.93 W/m^2^ and a resolution of 0.001 μW/m^2^. Data were recorded in MAX AVG (Average Smoothed) mode, following the methodology described in (Chu, 1974).

A 10 mL aliquot was placed in a 60 mm-diameter polystyrene Petri dish (Perint, Russia) and preheated to 37 °C using a PST-60HL-4 thermoshaker (Biosan, Latvia) for approximately 4 min. The sample temperature was monitored using a B. Well WF-4000 infrared thermometer (Switzerland).

**FIGURE 4:**
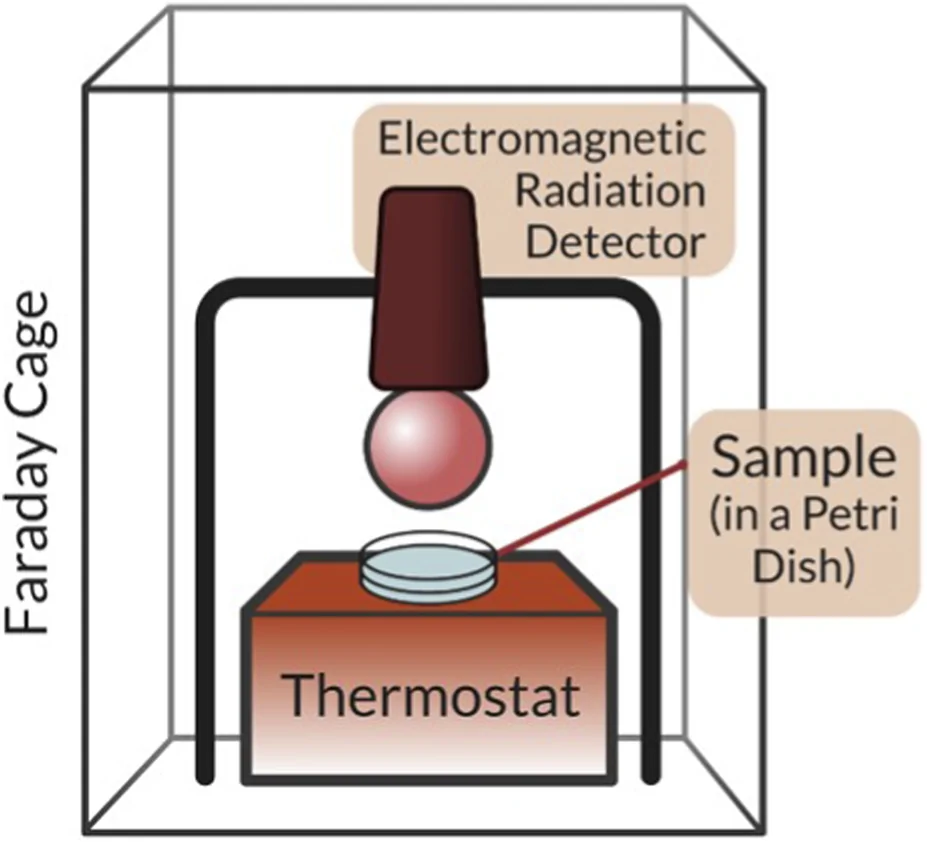
Experimental setup for radiometric measurements.

The experimental setup for radiometric measurements is shown in Figure 4. To provide electromagnetic shielding, the setup was placed in a Faraday cage made of an aluminum frame and a copper grid (with a 0.56 mm aperture). Inside the cage, a PL-H heating plate (Primelab, Russia) was used to maintain isothermal conditions and ensure uniform sample heating. The TES-92 EMI detector was mounted on a metal tripod. For radiometric data acquisition, the TES-92 detector was positioned 0.5 cm above the sample surface. Each measurement (repeated for three to five times) was conducted for 10 min, with the duration monitored by a TR 118 timer (Oregon Scientific, USA). After each measurement, the readings on the device display were saved, and the procedure was repeated sequentially for the rest of the samples.

### Radio-frequency spectroscopy: S_21_ transmission coefficient of aqueous solutions

**FIGURE 5:**
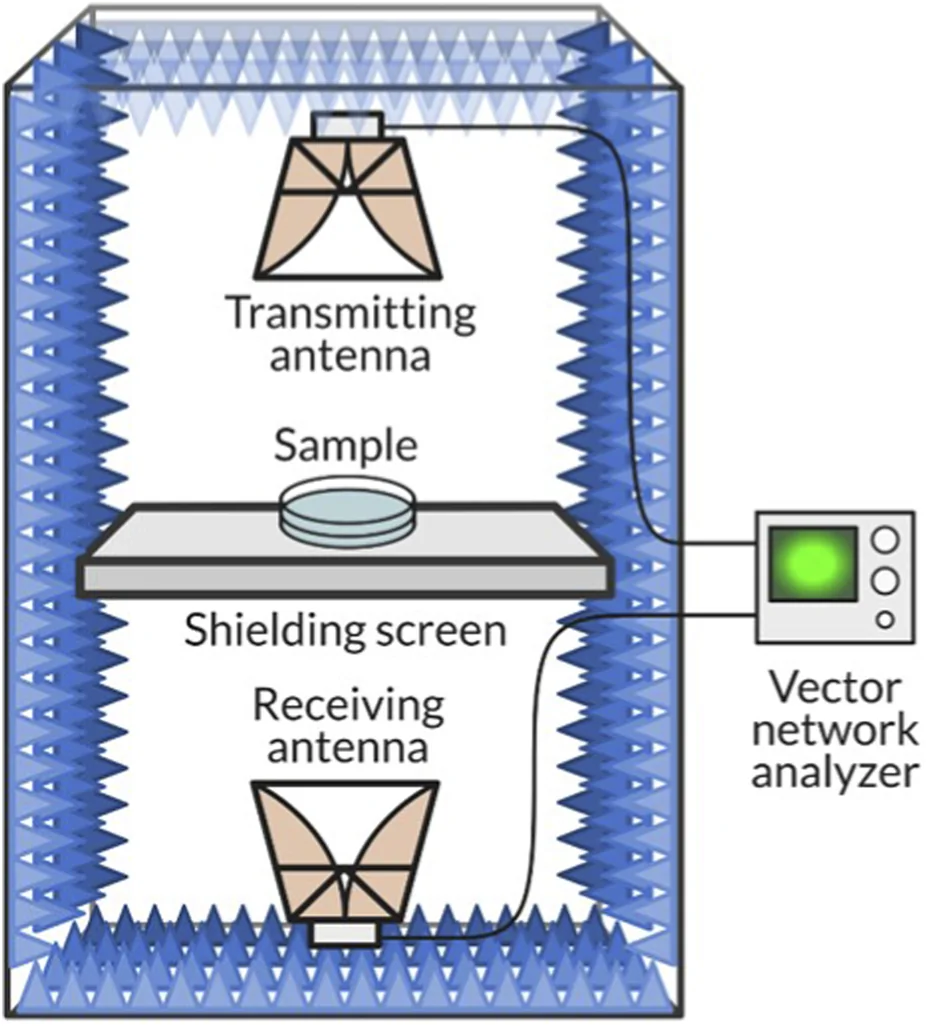
S_21_ radio-frequency spectroscopy setup.

The transmission coefficient (S_21_), which is a scattering parameter proportional to the ratio of transmitted to incident wave intensity, was measured using a Compact S5045 vector network analyzer (Planar, Russia). Two AIR-18 M horn antennas (Izmerilovka, Russia) served as the signal source and receiver. The transmitting antenna was positioned 25 cm above the sample. The sample itself was placed on a polystyrene foam base covered with RF-53 radio-shielding fabric, featuring an aperture for the sample container. This assembly was mounted directly above the receiving antenna (Figure 5).

To eliminate the external interference and minimize the internal reflections, measurements were conducted inside an anechoic chamber the casing of which was made of aluminum, while the internal lining, of Shtil 100-K10 radar-absorbing material (TRIM Measurement Systems, Saint Petersburg, Russia).

Measurements were carried out in a frequency range of 800 MHz to 2.8 GHz with a 10 MHz step at an output power of +5 dB m. A 10 mL aliquot of the sample was poured in a DURAN crystallizing dish (cat. no. 213133802, DURAN Group, Germany) covered with a polystyrene lid and placed on the measurement stage. To maximize the signal-to-noise ratio (SNR), data were acquired with an averaging factor of 999. For each sample, measurements were repeated 5–6 times.

It is worth noting that S_21_ coefficients characterize the changes in signal intensity as it passes through the medium, For this reason, these values were converted into values analogous to absorbance (optical density) using the following equation: A = –S_21_/10.

### High-resolution thermography

High-resolution thermal infrared (TIR) thermography was employed using a VarioCam HD head camera (InfraTec, Dresden, Germany) equipped with an IR 1.0/30 LW lens (Jenoptik, Jena, Germany). Data acquisition and image analysis were performed via IRBIS 3.1 Professional software (InfraTec, Germany). Aqueous samples (50 mL) were poured in polystyrene Petri dishes (Polyefir, Belarus) and preheated in a microwave oven (500 W for 90 s) to an initial temperature range of 53 °C–70 °C. For measurements, the dish was placed in a shielded enclosure with the camera mounted vertically above the sample. The dish lid was removed immediately before recording. Thermograms were recorded over a 25-min cooling period as the samples equilibrated toward an ambient temperature of 21 °C–23 °C. A representative fragment of thermal video imaging of a sample cooled down to approximately 30 °C is shown in the Discussion section. Detailed calibration procedure and acquisition parameters can be found in (Don et al., 2024).

### Statistical analysis

Statistical analysis was performed using the R environment (version 4.3.3; R Foundation for Statistical Computing, Vienna, Austria). Data normality and homogeneity of variances were assessed using the Shapiro-Wilk and Bartlett tests, respectively. For normally distributed data with equal variances, groups were compared using the Student’s t-test, while the Welch’s t-test was applied for unequal variances. Non-normal distributions were analyzed using the Mann-Whitney U-test (for two groups) or the Dunn’s test (for multiple comparisons). To account for multiple testing, p-values were adjusted using the Holm method. Effect sizes for pairwise group comparisons were quantified as Cohen’s d and interpreted according to conventional benchmarks (small, medium, large). Cohen’s d was calculated from the corresponding test statistics and sample sizes. Results are expressed as mean ± standard deviation (SD), and differences were considered statistically significant at p < 0.05.

## Results

**FIGURE 6:**
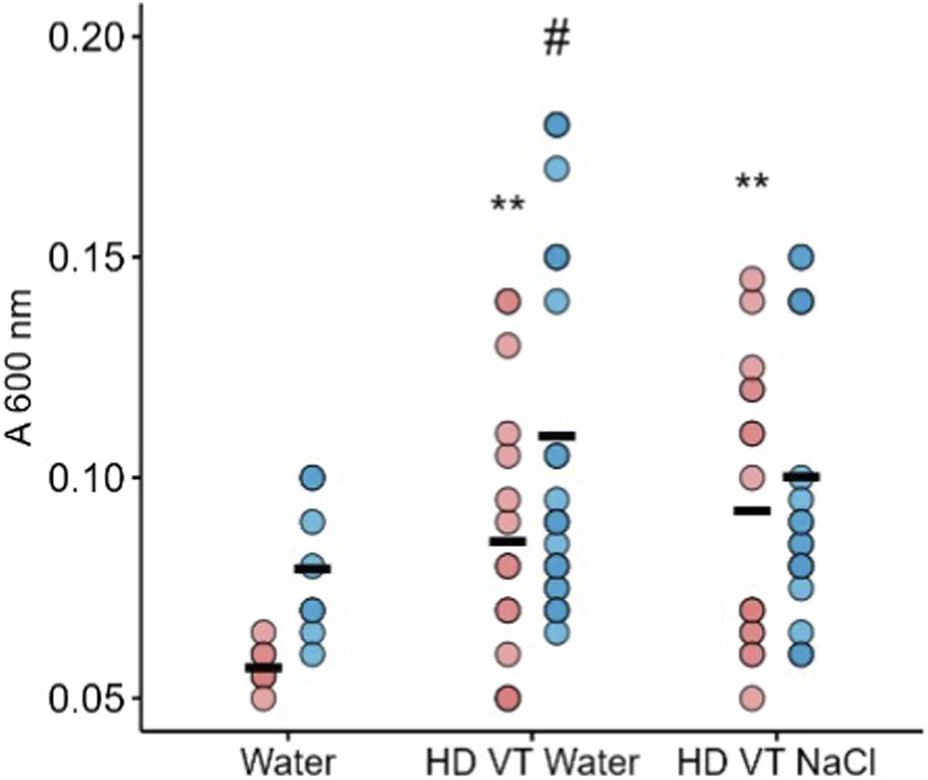
Changes in the absorbance of NaCl solution (10 mg/L) at a wavelength of 600 nm after an hour-long incubation in the outer samples. Blue markers represent the absorbance of a reference sample (“Water”), water subjected to intense vibrational treatment during successive dilutions (“HD VT water”), or NaCl solution subjected to intense vibrational treatment during successive dilutions (“HD VT NaCl”). Coral markers represent the absorbance of NaCl solution (10 mg/L) after an hourlong incubation in the outer samples: “Water”, “HD VT water”, and “HD VT NaCl”. Incubation was carried out according to the protocol shown in Figure 1. The results are given as mean ± standard deviation (n = 8–16). # p = 0.052 vs. Water (trend); **p < 0.01 (vs. inner sample incubated in Water), Dunn’s test. Effect sizes for blue markers: Water vs. HD VT water, d = 0.82 (large effect); Water vs. HD VT NaCl, d = 0.71 (medium effect); required n per group of 25 and 32, respectively. Effect sizes for coral markers: Water vs. HD VT water, d = 1.08 (large effect); Water vs. HD VT NaCl, d = 1.34 (large effect); required n per group of 15 and 10, respectively. Effect sizes were calculated as Cohen’s d. Sample size were calculated to achieve 80% power at α = 0.05.

Figure 6 shows the absorbance of the inner sample (NaCl solution, 10 mg/L) at 600 nm after an hourlong incubation in various outer samples (coral markers), compared to that of the outer samples before incubation (blue markers).

Outer samples were untreated water and water or NaCl solution subjected to repeated vibrational treatment during multiple dilutions. The absorbance of the inner sample before incubation was nearly identical to that after incubation in untreated water (reference sample) and, therefore, is not shown. Note that outer samples were not pre-irradiated with a high-energy electron beam before the incubation. The irradiation was always carried out immediately before the absorbance measurements. After incubation in vibrationally treated water or NaCl solution, the absorbance of the inner sample at 600 nm increased by nearly half (p < 0.01) compared to that after incubation in untreated water.

**FIGURE 7:**
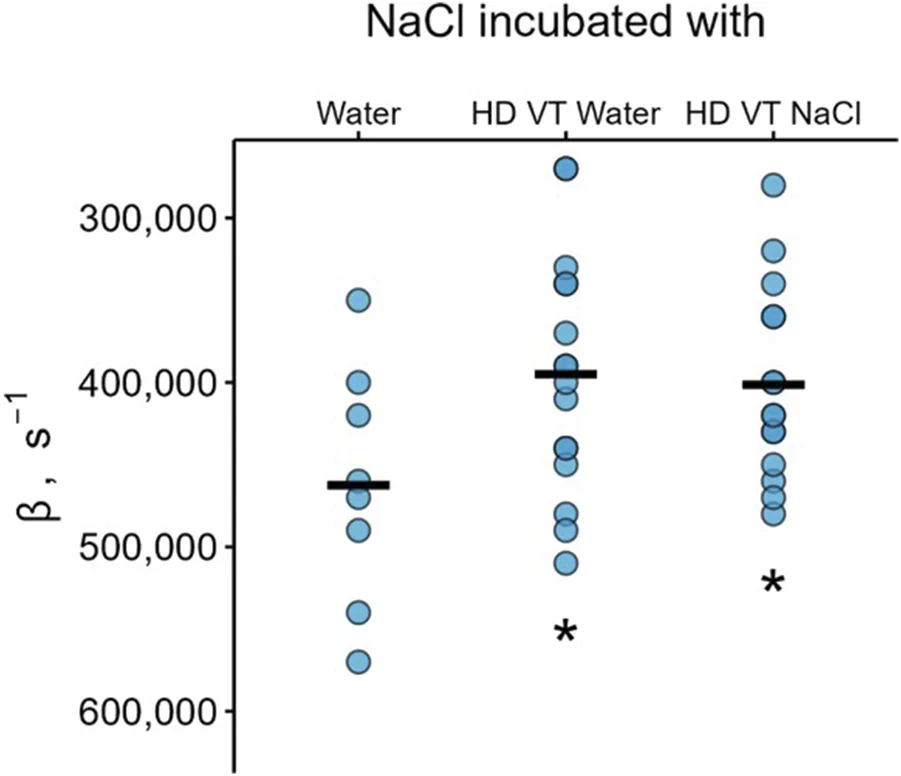
Change in the power of the exponent that approximates the absorbance (at 600 nm) decay in NaCl solution (10 mg/L) after an hour-long incubation in vibrationally treated outer samples. Outer samples were: “Water” (reference sample), water subjected to intense vibrational treatment during successive dilutions (“HD VT water”), and NaCl solution subjected to intensive vibrational treatment during successive multiple dilutions (HD VT NaCl). Results are given as mean ± standard deviation (n = 8–16). * p < 0.05 vs. inner sample incubated in “Water”, Student’s t-test. Effect sizes: Water vs. HD VT water, d = 0.93 (large effect); Water vs. HD VT NaCl, d = 0.96 (large effect); required n per group of 20 and 18, respectively. Effect sizes were calculated as Cohen’s d. Sample size were calculated to achieve 80% power at α = 0.05.

Previous work showed that secondary (hydrated) electrons (which are formed in the liquid phase upon the high-energy electron impact) can be localized within the inner surfaces of boundary hydration layers around the gas cores of nanobubbles after vibrational treatment (Novakovskaya et al., 2025). Therefore, in the present experiment, we also estimated the time dependence of the absorbance decay. Figure 7 shows the 600-nm absorbance decay (the exponent coefficient β in the *A*(*t*) = *e* ^−*βt*^ approximation) for the inner sample after the incubation in different outer samples.

Absorbance dropped most rapidly in the inner sample incubated in the reference sample (“Water”). Incubation in any vibrationally treated sample provided a much higher absorbance compared to incubation in the reference sample. Similar results, showing no principal differences among vibrationally treated solutions, were obtained by us previously (Novakovskaya et al., 2025).

These data suggest that vibrational treatment effects may be transmitted over a long range. Shaking the outer samples increases the bulk content of nanobubbles (Bunkin et al., 2025), and the nanobubbles affect the absorbance at 600 nm (Novakovskaya et al., 2025). Therefore, the incubation effect (i.e., the long-range influence of the outer samples on the inner one) likely promotes the nanobubble formation in the inner sample. This may be due to the emission of electromagnetic radiation by nanobubbles, which is the next topic of our investigation.

**FIGURE 8:**
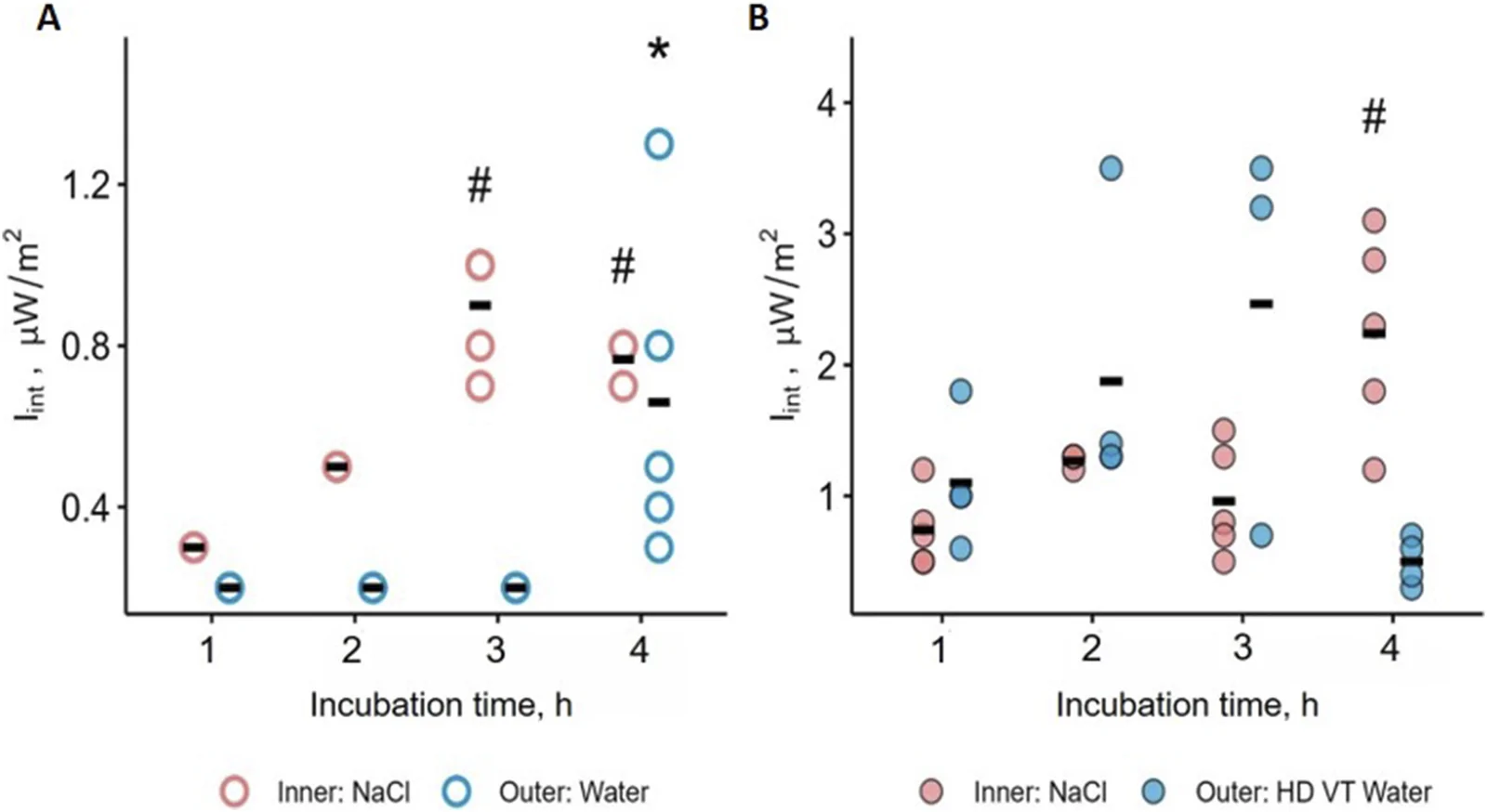
Intensity of the GHz radiation (I_int_) of NaCl solution (10 mg/L) incubated in outer samples for 1–4 h. **(A)** Coral empty markers represent the emission intensity of NaCl solution (“NaCl”) after 0–4-h incubation in an outer sample (untreated water, “Water”); Blue empty markers represent the emission intensity of “Water” outer sample after 0–4-h incubation with NaCl solution (blue empty markers). **(B)** Red filled markers represent the emission intensity of NaCl solution after 0–4-h incubation in an outer sample (water subjected to intense vibrational treatment during successive multiple dilutions “HD VT water”); Blue filled markers represent the radiation intensity of “HD VT Water” outer sample after 0–4-h incubation with NaCl solution. Results are given as mean ± standard deviation (n = 3–5). * p < 0.05 vs. “Water”, 0 h; # p < 0.05 vs. “NaCl”, 0 h, Dunn’s test. Effect sizes for blue empty markers: 1 h vs. 0 h; 2 h vs. 0 h (not applicable); 4 h vs. 0 h, d = 1.61 (large effect); required n = 8. Effect sizes for coral empty markers: 1 h vs. 0 h (not applicable); 2 h vs. 0 h, d = 5.61 (large effect); required n = 3; 4 h vs. 0 h, d = 1.61 (large effect); required n = 8. Effect sizes for blue filled markers: 1 h vs. 0 h, d = 0.91 (large effect), required n = 20; 2 h vs. 0 h, d = 1.30 (large effect), required n = 11; 4 h vs. 0 h, d = 1.58 (large effect), required n = 8. Effect sizes for coral filled markers: 1 h vs. 0 h, d = 2.22 (large effect), required n = 5; 2 h vs. 0 h, d = 0.61 (medium effect), required n = 44; 4 h vs. 0 h, d = 2.60 (large effect), required n = 4. Effect sizes were calculated as Cohen’s d. Sample size were calculated to achieve 80% power at α = 0.05.

Figure 8 shows the microwave radiation intensity (0.5–3.5 GHz) of the inner sample after incubation (1–4 h) in outer samples, compared to that of the corresponding outer sample. Outer samples were either untreated water (Figure 8A) or water subjected to repeated vibrational treatment during successive dilutions (Figure 8B). It is worth noting that results for both outer samples are close to the data obtained previously in a similar frequency range for the same samples after an hour of incubation and shown in Figure 6 in (Bunkin et al., 2025).

The data in Figure 8A can be treated as a reference, because both the inner and outer samples are pure water, which was not subjected to vibrational treatment, but was saturated with dissolved carbon dioxide (bicarbonate ions). Then, we can assume that both samples contain gas nanobubbles in an amount (see below) around 10^6^–10^7^ cm^−3^; and the sole difference between the samples is as follows: the inner sample is closed, while the outer one is open to the atmosphere. This means that the disturbing contact with the atmosphere affects the state of the outer sample solely. This is apparently manifested in the nearly unchanged level of radiation in the outer sample, whereas the radiation of the inner sample passes through a maximum in a range of 2–4 h.

Analysis of the results in Figure 8B reveals a non-monotonic change in the integral radiation intensity in both inner and outer samples. Note that the outer sample taken in this series of measurements was preliminarily subjected to repeated vigorous shaking. At certain time intervals, the intensity values were found to be nearly three times as high as the initial values (values before the incubation). Both samples demonstrate a trend of increasing intensity. Notably, after just an hourlong incubation (the very time interval that was used in the study of absorbance due to the existence of hydrated electrons generated upon the irradiation of samples with a high-energy electron beam), an increase in the GHz radiation intensity was observed. Similar changes can be noticed in the outer samples (Figure 8B, blue markers). The amplitudes of the intensity changes are approximately the same in the outer and inner samples, though the initial values in the vibrationally treated samples are nearly twice as high; and the temporal picture is characterized by a certain delay (about an hour-long) in the case of inner samples. The amplitude of the intensity change in the inner sample clearly matches that in the outer samples, which supports the hypothesis regarding the long-range effect.

**FIGURE 9:**
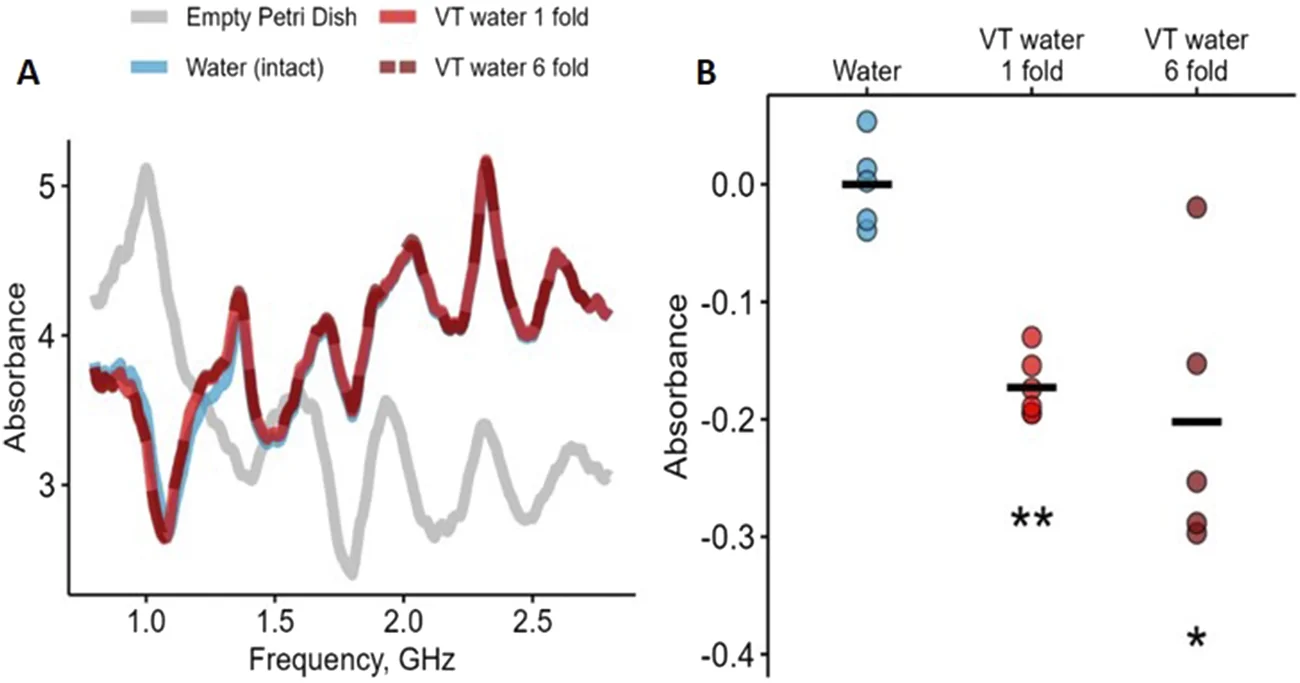
Absorbance at 0.8–2.8 GHz of intact water (“Water”), and water subjected to a single or 6fold vibrational treatment (“VT water”): **(A)** raw data and **(B)** integral data. As a reference, measurements with an empty Petri dish (panel A, gray curve) were used. All values are normalized by subtraction of the value of the “Water” (intact) sample. Data are given as mean ± standard deviation (n = 5–6). *p < 0.05 vs. “Water”, Welch’s t–test). Effect sizes: Water vs. VT water 1 fold, d = 5.50 (large effect); Water vs. VT water 6 fold, d = 2.32 (large effect); required n per group of 3 and 5, respectively. Effect sizes were calculated as Cohen’s d. Sample size were calculated to achieve 80% power at α = 0.05.

To clarify the role of the outer sample, measurements of the S_21_ parameters were also performed. This was done with a spectral sweep in a range of 0.8–2.8 GHz. The results are presented in Figures 9A,B.

The studied frequency range can be conditionally divided into two subranges, namely, 0.8–1.2 GHz dominated by Petri dish material absorption, and 1.2–2.8 GHz where the water absorption contributes significantly. In this setup, additionally to the reference water, we tested samples after single (1 × 20 shakes) and six-fold (6 × 20 shakes) vibrational treatment. Six-fold treatment produced a comparable to the single treatment but slightly more pronounced effect.

Overall, the vibration-induced absorption changes in the GHz range correlate with the changes in the emission intensity (Figure 8), implying the presence of some objects in the samples that both absorb and emit GHz waves. Oscillating objects, such as nanobubbles, whose boundary layers are charged due to the presence of anions (e.g., Cl^−^ or HCO_3_^−^), exhibit such properties.

## Discussion

If there are grounds to believe that secondary (hydrated) electrons generated upon the high-energy electron beam irradiation are localized within hydrogen-bond network defects, with enhanced stability in the case of localization within boundary/surface layers of nanobubbles (Novakovskaya et al., 2025), then, when interpreting the increased absorbance of the inner and outer samples one should take into account the nanobubble content (volume number density), apparent charges of their boundary layer, and their vibrational dynamics.

In an open cuvette with initially degassed water (under equilibrium conditions in the presence of atmospheric air dissolved in water, with a carbon dioxide content about 10^17^ cm^−3^), the bulk content of nanobubbles *n*_*b*_ in the presence of NaCl ions at a concentration of 10^–4^ – 10^–3^ M falls in a range of 10^6^–10^7^ cm^−3^ (Bunkin et al., 2016). Vibrational treatment (intensive mixing or hydrodynamic cavitation) should contribute to an increase in the *n*_*b*_ value due to vortex flows that disrupt liquid continuity, i.e., produce sufficiently extended defects in the hydrogen-bond network (Parmar and Majumder, 2013; Terasaka et al., 2011). Particularly, as our studies have shown, upon the vibrational treatment of water or NaCl solution at a frequency of 4 Hz and an intensity of 4 or 8 N, the bulk content of nanobubbles increases to 10^9^–10^10^ (Figure 1 in (Bunkin et al., 2025)). To further increase the nanobubble content, techniques such as spiral-liquid generation and cyclic pressure increase-decrease can be employed (Hu and Xia, 2018; Li et al., 2013; Pourkarimi et al., 2017).

At the same time, as shown in our previous work (Bunkin et al., 2025), oscillations of nanobubbles accompanied by the emission of GHz radiation can occur even in the absence of intense external influences, provided that convective flows exist in the aqueous solution. Below, parameters of convective flows in an aqueous solution are estimated, and their possible impact on the vibrational dynamics of nanobubbles, which can be accompanied by the emission of electromagnetic radiation in the GHz range, is analyzed.

Let us consider a 2-L flask filled with an aqueous solution and open to the atmosphere (see scheme in Figure 1), where surface evaporation of the liquid induces a temperature gradient. We assume that the height L of the liquid layer (i.e., the range of the temperature gradient predetermined by the surface evaporation) in the flask is ∼10 cm. A temperature gradient is known to cause various kinds of Rayleigh and Marangoni convective flows (Eggers and Villermaux, 2008; Landau and Lifshitz, 1960). The kinds of the resulting convective flows depend on the Rayleigh number, $Ra=\frac{g\beta ∆TL^{3}}{v\chi }$ , where *g* is the free-fall acceleration, *β* is the thermal expansion coefficient of water, Δ*T* is the temperature difference between the surface and the bulk water due to evaporation, *ν* is the kinematic viscosity of water, and *χ* is its thermal conductivity.

Let us estimate the Rayleigh number *Ra* for Δ*T* = 0.5 °C. Assuming that in our experiment *L* = 10 cm, we come to *Ra* = 5.8 × 10^6^. The Rayleigh number is usually compared to its critical value *Ra*_*crit*_; for water *Ra*_*crit*_ = 1100–1700. At *Ra* < 7.4 *Ra*_*crit*_ there is no convective flow in the liquid. At 7.4 *Ra*_*crit*_ < *Ra* < 9.9 *Ra*_*crit*_ laminar convection with a single circulation velocity arises. In a range of 9.9 *Ra*_*crit*_ < *Ra* < 11.01 *Ra*_*crit*_ laminar counter flows with different velocities exist. Finally, at *Ra* > 11.01 *Ra*_*crit*_ convection becomes turbulent. In our case, the threshold for turbulent convection is exceeded by a factor of approximately 10^3^.

**FIGURE 10:**
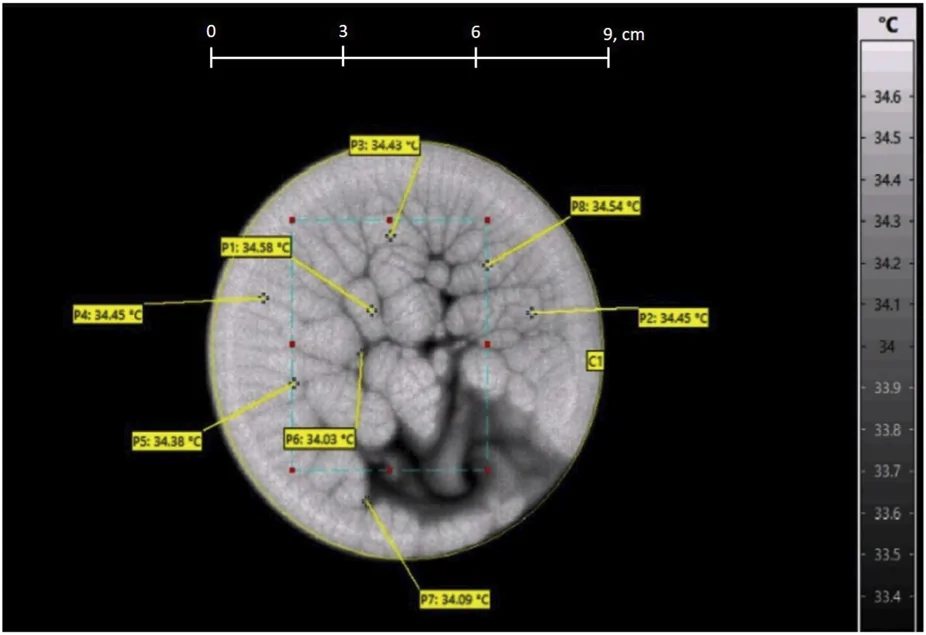
High-resolution thermograms of the surface of an aqueous solution. A centimeter scale bar is shown at the top of the image. Arrows mark temperatures at fixed points. On the right, the gray-gradient temperature scale is given.

Figure 10 shows high-resolution thermograms of heat flows in the surface layer of an aqueous solution, where the transverse dimension of this layer is 9 cm. These thermograms reveal a highly non-uniform temperature field in the layer, driving intense convective flows with temperature differences 2–3 times greater than the Δ*T* value used in the above estimates. Such flows may well not only promote the nanobubble formation but also cause deformations and oscillations of nanobubbles. As observed in the thermograms, and taking into account that the transverse dimension is 9 cm, the characteristic transverse scale of the resulting turbulent flows is approximately 1 cm. Since the liquid layer thickness is 10 cm, it can be concluded that the turbulent flows are elongated along the vertical axis, and these flows should entrain a significant number of gas nanobubbles.

Irradiation of samples with high-energy electrons induces additional strong medium perturbations, promoting nanobubble formation along electron tracks. Furthermore, if the outer sample (see scheme in Figure 1) was subjected to vibrational treatment, its initial nanobubble content was already high. Previous results indicate that secondary electrons, which appear upon the high-energy electron impact, can be localized efficiently within nanobubble spherical boundary layers (Novakovskaya et al., 2025). In NaCl solution, this effect should be less pronounced because of the structural similarity of the domains, where excess electron and chloride ion can be localized (Novakovskaya et al., 2025).

As shown in Figure 6, incubating NaCl solution (inner sample) in an outer solution with a high content of nanobubbles (VT water or HD VT water) causes an increase in the absorbance at 600 nm upon the high-energy electron irradiation. Although absorbance remains lower than in the outer samples (which is expected because of the high chloride ion content), the relative increase (∼40%) nearly matches that previously observed in the samples subjected to strong vibrational treatment (Novakovskaya et al., 2025). Similar results were also obtained for the absorbance decay (Figure 7), where the changes in the inner sample were comparable to those in the vibrationally treated outer samples (Novakovskaya et al., 2025). According to the previously proposed concept (Novakovskaya et al., 2025), these changes in the absorbance arise from the reversible localization of secondary electrons in nanobubble boundary layers. This raises a question about the mechanism that may be responsible for the generation of nanobubbles in the untreated inner sample, which was incubated in close proximity with the vibrationally treated outer sample (separated by the vial wall)?

### Oscillations and radiation of nanobubbles

According to our model concept, nanobubble can be considered as a gas core surrounded by a spherical boundary layer (at least trimolecular in thickness) where anions (chloride ions in NaCl solution) are predominantly localized (Bunkin and Bunkin, 2016; Bunkin et al., 2016; Bunkin et al., 2025; Yurchenko et al., 2016). Some cations (sodium ions or hydrated protons) can also be localized in this layer, but the majority of the counterions are localized in the diffuse layer around the nanobubble.

**FIGURE 11:**
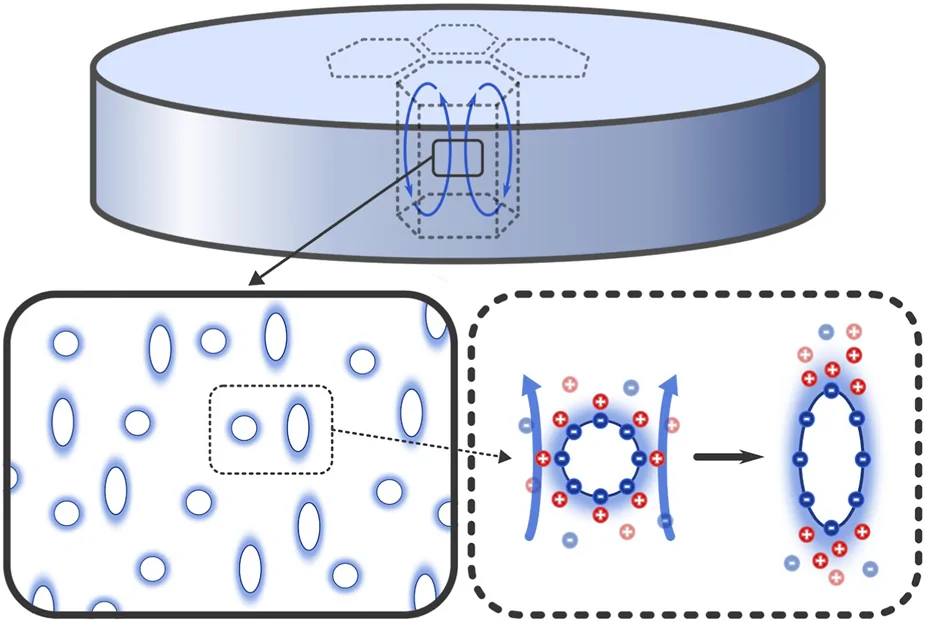
Schematic representation of the effects of turbulent hydrodynamic flows around a negatively charged nanobubble surrounded by a layer enriched in counterions (cations). The boundary layer of the nanobubble where anions are localized. A spherical nanobubble surrounded by a diffuse counterion-containing layer with arrows indicating turbulent hydrodynamic flow directions. The transformation into an ellipsoid driven by a stretching Coulomb force arising due to the elevated cation density at the poles and reduced density at the equator.

We propose that intense turbulent hydrodynamic flows can cause a redistribution of the ion density *ρ(r)* within the screening diffuse layer that involves counterions. The Poisson-Boltzmann equation solved for nanobubbles assumes complete isotropy (no polar or azimuthal angle dependence) in the radially symmetric *ρ(r)* ion density distribution in the liquid (see (Bunkin et al., 2025) for more detail). However, the *ρ(r)* density redistribution in the diffuse layer can give rise to a stretching Coulomb force and the resulting change in the nanobubble shape from spherical to ellipsoidal. This transformation is illustrated schematically in Figure 11. Figure 11 (top part) shows water flow directions (arrows) near a nanobubble surrounded by a screening diffuse layer. Anions localized in the spherical boundary layer can be considered as fixed within the H-bond network of water, whereas counterions in the diffuse layer outer to the Stern layer (Stern, 1924) can be shifted by the hydrodynamic flows due to viscous forces. Figure 11 (bottom part) depicts a possible scenario: the local content of cations decreases near the nanobubble equator but increases close to the poles, generating a Coulomb force that stretches the nanobubble. This leads to a change in the shape of the nanobubble from spherical to ellipsoidal, while its volume being preserved. In this case, the shape of the bubble tends to be restored due to the surface tension forces, which should result in the oscillations of the nanobubble. This mechanism of oscillation generation is fundamentally different from the activation of radial oscillations of nanobubbles by a sound wave, which is described by the Rayleigh–Plesset equation ((Hongray et al., 2014), as well as the comments to Equation (9) in (Bunkin et al., 2025)). Indeed, when a bubble is excited by a sound wave in the negative phases of sound pressure, the bubble radius increases. In this case, the spherical shape of the bubble is preserved, but its volume changes.

Thus, turbulent hydrodynamic flows create conditions for efficient shape modification of nanobubbles due to the redistribution of the screening charge density between the poles and the equator. The transformation of a spherical nanobubble into an ellipsoid and back should lead to the excitation of its resonant oscillations.

The Stern layer, which includes counterions and is tightly adjacent to the gas core of the nanobubble, cannot be washed away by convective flows. Consequently, in this case we deal with a compound particle, namely, a gas core inside a charged spherical shell surrounded by the Stern layer. The size of the compound particle is *a*_*c*_ ∼ *R*_*0*_ = 100 nm with a net charge of *Q*_*c*_ ∼ 60 *e*, where *e* is the elementary charge (see (Bunkin et al., 2025)); this estimate is based on the findings of (Ulatowski et al., 2019). We further assume that the bulk content of compound particles is on the order of *n*_*b*_ ∼ 10^10^ cm^−3^ (see above), which corresponds to an average distance between two neighboring compound particles of <*r*>∼ 4.5 µm.

Insofar as the characteristic spatial scale of a hydrodynamic vortex that causes the redistribution of ions within the diffuse layer is on the order of πL (where L = 10 cm is the thickness of the liquid layer), nearly all compound particles within a 1 cm^3^ volume are captured by the vortex. If we assume that all nanobubbles initially had similar spherical shapes (Bunkin and Bunkin, 2016; Bunkin et al., 2016; Yurchenko et al., 2016) and that the vortex acts equally on all nanobubbles across the πL scale, then the compound particles modified by the redistribution of peripheral ions in the diffuse layer will transform into similar ellipsoids (as illustrated by Figure 11). Furthermore, taking into account that the geometric parameters of the ellipsoid determine the initial phase of oscillations, and assuming that all the resulting ellipsoids are similar, we can expect that the oscillations of a sufficiently large number of such ellipsoids occur at the same frequency and with synchronized initial phases. Consequently, the resulting oscillations will be coherent. And the number of coherent oscillators should increase upon the vibrational treatment of the sample.

The redistribution of peripheral ions within the diffuse layer occurs during the motion of the compound particle. Let us assume that this redistribution takes place rapidly; thus, within the corresponding (short) time interval τ, the motion of the compound particle can be considered as a straight-line with a sufficient accuracy, which makes the local laminar approximation valid. In this case (see (Landau and Lifshitz, 1960) for more detail), for the redistribution of the peripheral segments of the diffuse counterion-containing layer to occur, the electrostriction pressure $p_{str}=\frac{Q_{c}^{2}}{{8\pi \varepsilon a}_{c}^{4}}$ on the surface of the compound particle should be equal to the maximum pressure of the detachment (shift) of peripheral ions $p_{v}=\frac{3{\eta u}_{0}}{{2a}_{c}}$ , where *u*_*0*_ is the velocity of laminar motion of a compound particle with radius *a*_*c*_ and charge *Q*_*c*_, *η* is the dynamic viscosity of water, and *ε* = 82 is its dielectric permittivity. In deriving this formula, the Stokes friction force was estimated as *F = 5ηa*_*c*_ *u*_*0*_, which corresponds to the motion of a liquid droplet with radius *a*_*c*_ and viscosity *η* in a liquid of the same viscosity (Landau and Lifshitz, 1960). Given *a*_*c*_ = 100 nm, *Q*_*c*_ = 60e, from the condition $\frac{3\eta u_{0}}{{2a}_{c}}=\frac{Q_{c}^{2}}{{8\pi \varepsilon a}_{c}^{4}}$ we obtain a velocity estimate of $u_{0}=\frac{Q_{c}^{2}}{{12\pi \varepsilon \eta a}_{c}^{3}}=3.2\text{ mm}/s$ , which is necessary for the displacement of the peripheral ions of a diffuse layer. Judging from the thermal video imaging, the screenshots of which were shown in Figure 10, the velocities of vortex flows in the surface layer of a sample contained in a Petri dish reach 5 cm/s, being an order of magnitude higher than the obtained theoretical estimate.

For the redistribution of peripheral ions to occur, a composite particle should change its position in a viscous fluid by a distance approximately equal to its radius *a*_*c*_. Such a displacement may be regarded as a straight line, which justifies the use of the laminar approximation. The time required for the redistribution of peripheral ions within the diffuse layer can be estimated as follows: *τ* ≤ *a*_*c*_/*u*_0_ = 2 × 10^−5^ s. The short-term deformation of the diffuse layer caused by the shift of peripheral ions from the equator to the poles of a compound particle is analogous to the action of a Coulomb force impulse in a *τ* time interval. Such an impulse excites oscillations of the compound particle at its resonant frequency.

Insofar as the velocity of vortex flows generated by turbulent convection is non-uniform in the bulk liquid, a compound particle may enter a region where the vortex velocity equals *u < u*_0_. In this case, the uniform counterion distribution within the diffuse layers of compound particles should be restored (Figure 11). If later on the compound particle again finds itself in a region where the vortex velocity meets the condition of *u ≥ u*_0_, the peripheral layers of the diffuse cloud should again be disturbed. This causes a redistribution of the ions within the diffuse layer; in other words, whenever the condition *u ≥ u*_0_ is met, the compound particle is affected by another Coulomb force impulse, which promotes its deformation (Figure 11).

Thus, we can describe this process as a random sequence of Coulomb force impulses. An average period *T*_0_ of the impulses is not precisely known, but still let us assume that the condition *T*_0_ >> *τ* = 2 × 10^−5^ s is met, which means that the period exceeds the characteristic time required for a significant redistribution of peripheral ions. In other words, the *T*_0_ time interval is sufficient for restoring the volume density distribution of ions. Therefore, for certainty, we assume *T*_0_ ∼ 10^–4^ s.

Previously, we estimated the resonant frequencies of radial oscillations of nanobubbles to be ω_0_ ≈ 10^9^ Hz with a characteristic decay time of *τ*_0_ = 1/β ≈ 4 μs, as well as the oscillation frequencies driven by the thermal motion of molecules in the environment characterized by ellipsoidal distortions of nanobubbles (ω_n0_ = 2.7 × 10^9^ Hz) (Bunkin et al., 2025). It is evident that these negatively charged oscillating compound particles can emit electromagnetic waves.

The intensity of such radiation at an assumption of the co-presence of individual nanobubbles and their coalesced aggregates was also estimated previously (Bunkin et al., 2025). It is worth noting that the model used was based on the earlier proposed concept of the oscillations of charged water droplets (Grigor’ev et al., 2015). The decay of such oscillations is due to the emission of electromagnetic waves related to the oscillations; in a gas medium, this is naturally the sole mechanism for the attenuation of oscillations.

However, oscillations of the charged compound particles analyzed here occur in a viscous environment. The decay time of these oscillations is *τ*_0_ ≈ 4 μs (see above), and the oscillations arise as a result of random Coulomb force impulses with an average period of *T*_0_ ∼ 10^–4^ s. Hence, when evaluating the generation of electromagnetic waves due to the excitation of compound particles, it is necessary to account for the intermittency factor, which is determined by the ratio *τ*_0_/*T*_0_ = 4 × 10^−2^. This requires a modification of the equation, which was used in (Bunkin et al., 2025) for estimating the resulting radiation intensity:

$$I=\frac{\tau_{0}Q^{2}}{{4T}_{0}\varepsilon \left(\omega_{n0}\right)}\frac{\Gamma^{n+1}}{c^{2n+1}R^{n+5}}\beta_{n}^{2}{\left[\frac{1}{\left(2n-1\right)‼}\right]}^{2}\frac{{\left(n+1\right)}^{n+3}(\left(n-1\right){\left.\left(n+2\right)\right)}^{n+1}\rho_{g}}{\rho_{l}^{n+2}n^{2}\left(2n+1\right)}.$$

This expression represents the radiation intensity of a single compound particle with a charge *Q*; $c=\frac{c_{0}}{\sqrt{\varepsilon }}$ where *c*_0_ is the velocity of light in vacuum, Г = 73 dyn/cm is the surface tension coefficient at the gas–water interface, *ρ_l_* = 1 g/cm^3^ is the density of water, and *R* = *R*_0_ = 100 nm is the radius of the nanobubble. The coefficient *β*_*n*_ ∼ Δ*R*/*R*_0_ ∼ 0.01 is a ratio of the deformation Δ*R* of a compound particle to its unperturbed radius *R*_0_ at a constant total volume of the compound particle. For *n* = 2, ω_*n0*_ = 10^9^ Hz, ε(ω_*n*0_) = 82, we obtain the radiation intensity of a single compound particle with a charge *Q* ≈ 60 e (this estimate follows from the study of (Ulatowski et al., 2019), where the ζ-potential was measured to be −10 mV for nanobubbles with a 100 nm radius) that equals I_1_ ≈ 1.6 × 10^−33^ W/m^2^.

In (Bunkin et al., 2025) it was suggested that some objects identified in DLS experiments with apparent radii of 75–100 nm are actually clusters (aggregates) of smaller nanobubbles, which are characterized by different radiation intensities. If a bubble’s radius is smaller by half, its charge (at the same density) should be smaller by a factor of four. Hence, the *Q* ^2^/*R* ^7^ ratio increases eightfold, which corresponds to an intensity of I ≈ 1.3 × 10^−32^ W/m^2^ (at an almost thrice as high frequency).

Additional stabilization of such aggregates is provided by the ellipsoidal deformation of individual nanobubbles in the Coulomb force field induced by convective flows. Electrostatic interactions of charged polarized ellipsoids are expected to predetermine the in-phase alignment of their ellipsoidal oscillations, resulting in the coherence of oscillations within the nanobubble ensembles. Assuming the ratio of the individual to cluster nanobubble radius to be 1:2, a cluster of bound nanobubbles should involve approximately 10 aggregated nanobubbles. The integral radiation intensity of such a cluster is then I_2_ = 10^2^ I ≈ 1.3 × 10^−30^ W/m^2^.

Upon vigorous shaking, the concentration of nanobubbles is *n*_b_ = 10^10^ cm^−3^ (as shown above). To a first approximation, we assume that individual nanobubbles are similar on average (and their fraction is *n*_1_), while nanobubble clusters of the same visible size are composed of nanobubbles approximately half as large (and their fraction is *n*_2_). Then the bulk contents of individual nanobubbles and their clusters equal 10^10^ *n*_1_ and 10^10^ *n*_2_ cm^−3^ respectively (where *n*_1_ + *n*_2_ = 1). Consequently, the average distance between nanobubbles of both types is approximately 4.5 µm. Insofar as they are located in the same field of convective hydrodynamic flow (as discussed above), they move in a consistent way, and their initial oscillation phases are identical. This synchronization leads to the formation of two coherent ensembles of emitting oscillators.

Some clarification is necessary here. The assumption of in-phase (coherent) oscillations of individual nanobubbles/their clusters is purely intuitive and is based on the results of the thermographic experiment. As shown in the thermogram in Figure 10, the characteristic transverse scale of the convective flow is approximately 1 cm, and the liquid layer depth is 10 cm, meaning that we are dealing with convective flows elongated with respect to the vertical axis. Since the density of nanobubbles and their clusters is quite high (approximately 10^10^ cm^−3^), and the average distance between these particles is approximately 4.5 µm, convective flows capture a significant number of nanobubbles and their clusters. Moreover, at scales of approximately 1 cm, the laminar approximation can be used, implying it can be assumed that the motion of nanobubbles within a volume of approximately 1 cm^3^ is steady and straight-line. Assuming further that the nanobubbles and their clusters, captured by the convective flow, are identical to one another, we can postulate that the oscillations of these particles will be excited with the same phase, meaning that we are dealing with a coherent ensemble of emitting particles. Obviously, the dephasing of these oscillations occurs when a nanobubble/cluster leaves the convective flow. As shown earlier, we believe that the oscillations of nanobubbles/clusters arise as the result of a random sequence of Coulomb force pulses. Thus, the duration of such a pulse can be conventionally considered as the time interval during which the coherent properties of the oscillator ensemble are preserved. According to estimates, the duration of such a pulse is *τ* = 2 × 10^−5^ s, and the random repetition period of such pulses exceeds this duration (we estimated this period to be *T*_0_ ∼ 10^–4^ s, see above), meaning that the oscillations become dephased in the intervals between pulses, and coherence is disrupted. Since the oscillations of the nanobubbles completely decay within the time *τ*_0_ = 4 μs << *T*_0_, then the ensemble of emitters is at rest by the next pulse of the Coulomb force, implying that for each new pulse, the conditions for the coherent excitation of oscillations of the nanobubbles/clusters are reproduced.

Assuming coherence of emitting oscillators, the total radiation intensity within a 1 cm^3^ volume can be estimated as I_0_ = (10^10^ n_1_)^2^ I_1_ + (10^10^ n_2_)^2^ I_2_ ≈ (n_1_)^2^ 1.6 × 10^−13^ + (n_2_)^2^ 1.3 × 10^−10^ (W/m^2^). Recall that the volume of the outer liquid sample involved in the incubation process is 2 L (see Figure 1). Under the assumption that the compound particles oscillate coherently within a 1 cm^3^ volume, the total radiation intensity generated in the 2-L volume of the outer sample is as follows: I_compl_ ∼ (n_1_)^2^ 3.2 × 10^10^ + (n_2_)^2^ 2.6 × 10^−7^ (W/m^2^).

These estimates provide a plausible explanation of the observed features of radiation emission and absorption in the GHz range, as well as the absorbance decay at 600 nm upon the high-energy electron beam impact. This explanation holds provided that there exists a mechanism that predetermines the transmittance of radiation from the outer sample to the inner one and its resonant absorption (by the inner sample), which causes an increase in the bulk content and stabilization of nanobubbles in the inner sample.

In undisturbed water, radiation in the GHz range is virtually unabsorbed. As is well established, the interaction of an external electromagnetic wave with an environment is described by the complex permittivity ε = ε′ − iε″, where the real part ε′ represents the “ability” of the medium to be polarized by an external field, while the imaginary part ε″ describes the energy losses caused by the absorption and conversion to heat (Angulo-Sherman and Mercado-Uribe, 2011; Landau and Lifshitz, 1960). For the ε′ and ε″ coefficients we have $\varepsilon^{\prime }\left(\omega \right)=\frac{\varepsilon_{s}-\varepsilon_{\infty }}{1+\left(\omega^{2}\;\tau^{2}\right)}$ + $\varepsilon_{\infty },\varepsilon^{\prime \prime }\;\left(\omega \right)=\frac{\left(\varepsilon_{s}-\varepsilon_{\infty }\right)\left(\omega \tau \right)}{1+\left(\omega^{2}\tau^{2}\right)}$ , where τ is the rotational diffusion time of water molecules (Landau and Lifshitz, 1960): $\tau =\frac{{4\pi \eta r}^{3}}{kT}$ , ε_S_ is the static permittivity $\left(\text{for water }\varepsilon_{S}=82\right),\varepsilon_{\infty }$ is the permittivity in the optical range $\left(\varepsilon_{\infty }=1.77\text{ for water}\right)$ , r is the radius of the molecule (r = 1.38 Å for water), η is the dynamic viscosity (η = 8.9 × 10^−4^ Pa s for water). For water under normal conditions, we have τ ≈ 3.3 ps. This leads to ωτ ≈ 3 × 10^−3^, which means that ε″ << ε' ≈ ε_S_, and at low radiation intensities in this frequency range, thermal effects caused by the absorption may be neglected. Hence, the radiation emitted by nanobubbles in the outer sample (see Figure 1) as a result of the vibrational treatment should efficiently reach the inner sample.

Note that the rotational relaxation time of water molecules increases significantly close to the surfaces of the flask, at both the outer and inner sample interfaces. For instance, on a silicate surface, the relaxation time is 4 to 5 times as large as in the bulk liquid, depending on the specific crystallographic face (Agarwal et al., 2023). Under these conditions, radiation absorption becomes appreciable and can facilitate the formation of additional nanoscale defects within the hydrogen bond network of the water molecules. It should also be noted that, according to the measurements performed, an empty glass Petri dish also absorbs radiation in the GHz range (Figure 9A), which should induce its oscillations and, hence, facilitate the alike deformations of the water layer adjacent to its other side that faces the inner sample. These deformations can act as nuclei of nanobubbles in the inner solution. Given that the surface area of the flask filled with the inner sample is about 290 cm^2^, the radiation intensity resonantly transmitted to the inner sample from the outer sample can be estimated as at least 0.1 nW. This intensity level is sufficient to promote the formation of defects, at least within the interfacial region of the inner solution adjacent to the flask walls.

**FIGURE 12:**
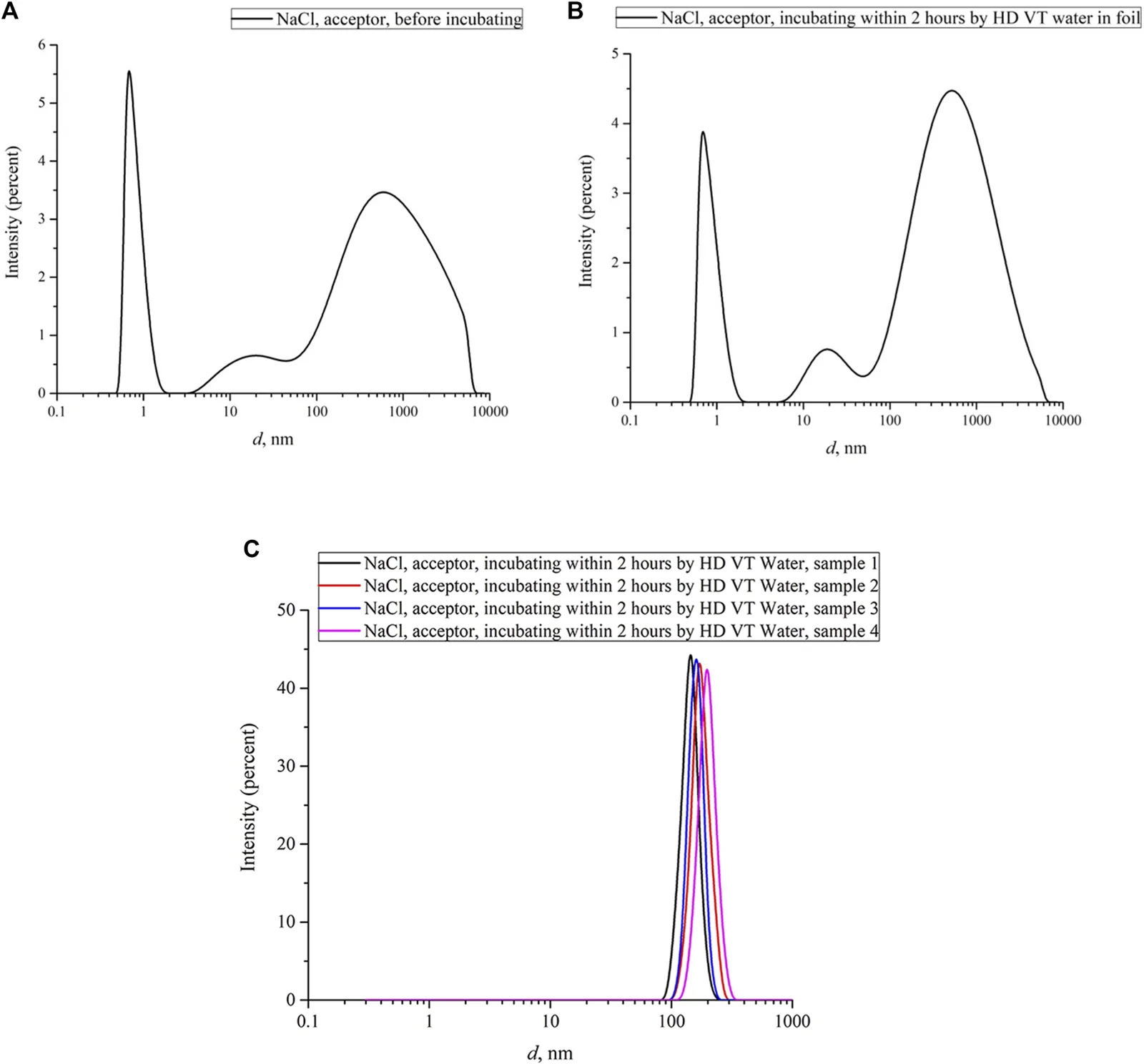
Size distributions of scattering particles in a NaCl solution (A) before incubation; (B) after incubation when the vial with the sample was coated with an aluminum foil with a thickness of 10 microns; and (C) after incubation for 2 h in HD VT water

To test this hypothesis, we performed DLS experiments for the inner sample (aqueous NaCl solution, 10 mg/L) both before and after a 2-h incubation in an outer “HD VT water” sample (Figure 12). This particular sample was selected because the incubation of the inner NaCl solution with vibrationally treated outer samples leads to a measurable increase in the GHz radiation intensity of the inner solution (Figure 8). Additionally, in several DLS experiments, NaCl solution samples were coated with an aluminum foil to shield them from electromagnetic radiation. In the latter cases, the amplitude of electromagnetic waves at a given frequency should attenuate when penetrating in a conducting medium due to the skin effect (Landau and Lifshitz, 1960). The depth of the skin layer Δ calculated in the SI system can be estimated as follows: $∆=503\sqrt{\frac{\rho m}{\mu_{m}f}}$ , where *ρ*_*m*_ is the specific resistance of the metal, µ_*m*_ is the relative magnetic permeability (close to unity for diamagnetic and paramagnetic materials), and *f* is the frequency in Hz. For the purpose of our estimation, we consider an aluminum screen. Using the parameters for aluminum $\left(\rho_{m}=2.7\times 10^{-8}\;\Omega \times m,{µ}_{m}=1\right)$ and a resonant frequency of nanobubbles (ω_res_ ≈ 4 × 10^9^ rad/s), we obtain a frequency *f* = 6 × 10^8^ Hz. This gives an estimated skin depth of ∆ = 3 µm. In our experiments, the incubated inner NaCl solutions were coated with an aluminum foil with a thickness of 10 µm. If the incubation effect is indeed driven by electromagnetic radiation from gas nanobubbles oscillating at their resonant frequency, then shielding the sample with the foil should efficiently level off the effect. The results of these measurements are shown in Figure 12.

As shown in Figure 12, upon the incubation of the inner solution, the size distribution of scattering objects reflects a significant content of the objects with diameters ranging from 146 to 196 nm, which are typical hydrodynamic diameters of nanobubbles.

**FIGURE 13:**
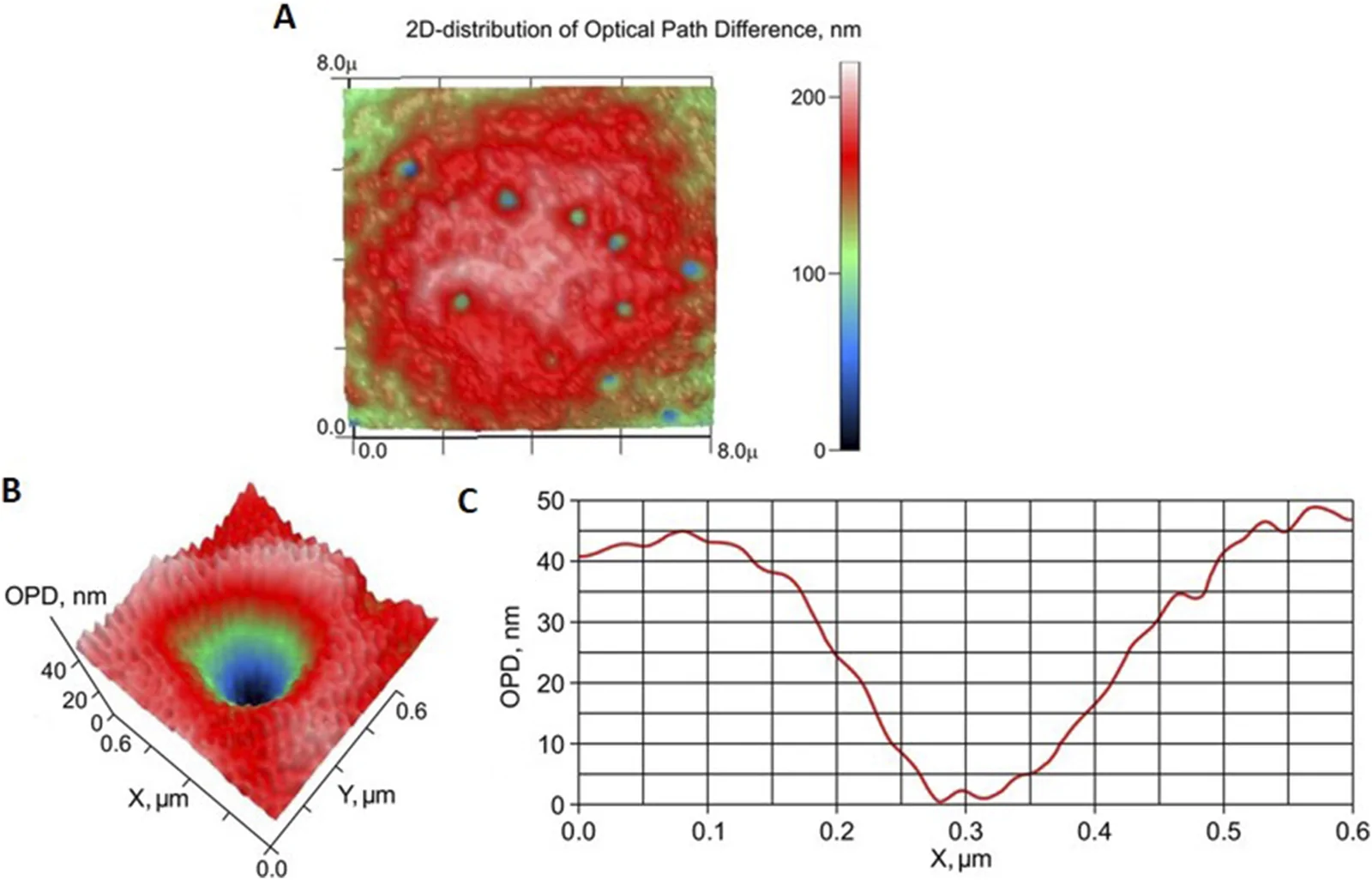
Results of LPM experiments for gas nanobubbles. Panel **(A)** colormap of 2D distribution of the optical path difference (OPD) of a sample area (8 × 8 μm^2^); objects of 200–300 nm in size, for which the OPD distribution has a concave profile, are clearly visible. Panel **(B)** 2D distribution of OPD in the vicinity of an object with a concave OPD; Panel **(C)** 1D profile of OPD across the object shown in panel **(B)**; according to the calibration data, the refractive index of this object is *n* ≈ 1.03, indicating it is a gas nanobubble.

Note that when studying nanobubbles, it is necessary to combine two experimental techniques: such as DLS (which can be used to establish the existence of scatterers of a certain size in a liquid sample and even, in some cases, to determine the bulk density of these scatterers) and laser phase microscopy (LPM), which can not only determine the size of scatterers but also allows to find their optical density (Nirmalkar et al., 2018). Indeed, in DLS experiments, the effect of scattering occurs because the refractive index of the scattering particle differs from that of the ambient liquid, i.e., in this case, the absolute value of the refractive index difference plays a role. In these experiments, it is fundamentally impossible to distinguish a solid nanoparticle from a gas nanobubble. In LPM the refractive index of the particles can be determined, i.e., it is possible to discriminate between particles with a high refractive index (e.g., solid-state nanoparticles) and gas nanobubbles. Figure 13 shows 2D distribution of OPD and its 1D profile along the X-axis for an individual particle with a concave profile and a size of 300 nm, respectively. According to equation $\Delta h=\frac{\delta \cdot \lambda }{2\pi \alpha }=\frac{d\cdot \Delta n}{\alpha }$ , see the comment in Laser Phase Microscopy, the concave profile of OPD means that the refractive index of the displayed particles is lower than that of the surrounding liquid. An estimate of the refractive index of these particles gives *n* ≈ 1.03, indicating that these particles should be gas nanobubbles (Bunkin et al., 2021; Nirmalkar et al., 2018).

**FIGURE 14:**
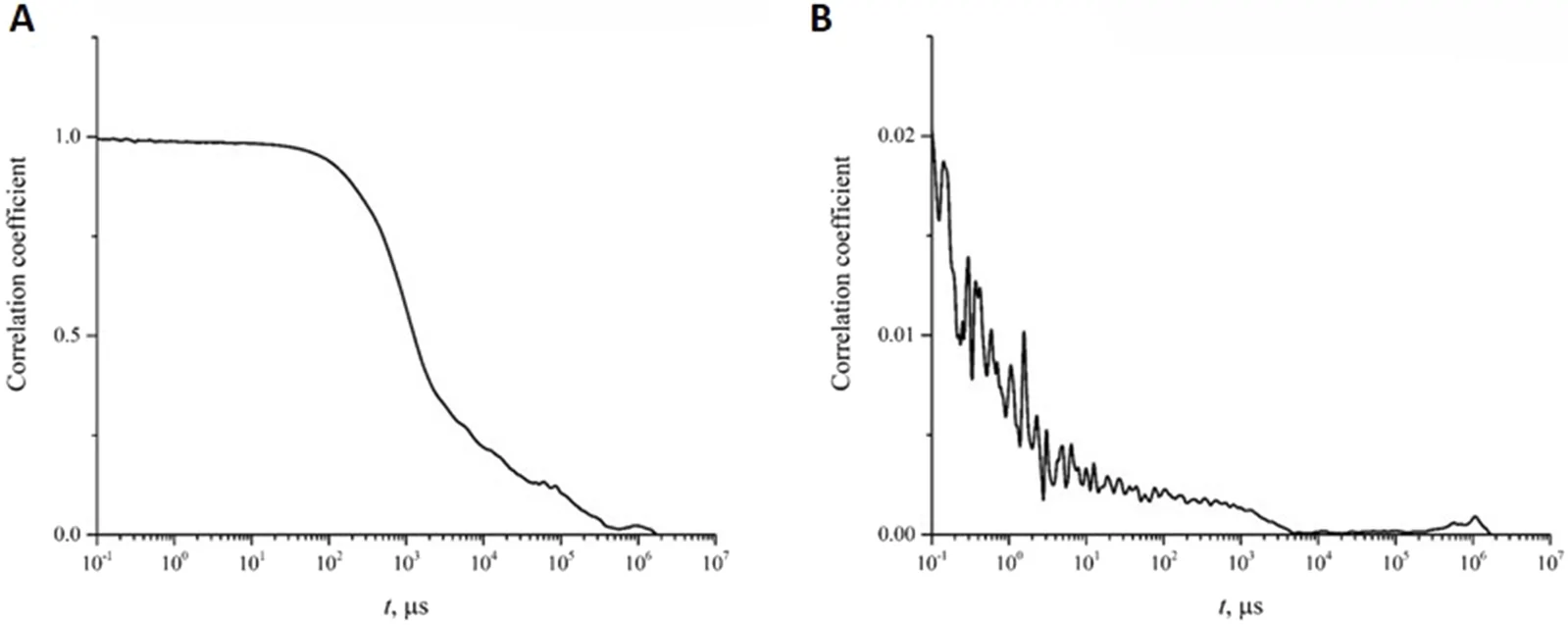
Correlation coefficients for NaCl solution **(A)** after the incubation in HD VT water and **(B)** after incubation when the vial was coated with an aluminum foil with a thickness of 10 μm. See text for details.

By contrast, the same samples prior to incubation were characterized by a broad unspecific size distribution with no pronounced peak in the range of 100–200 nm. It is worth noting that the correlation coefficient for the incubated samples (Figure 14A) was two orders of magnitude higher than that for the initial samples or those incubated in aluminum foil (Figure 14B).

Furthermore, for the incubated (unshielded) samples, the correlation coefficient at time t = 0 was equal to unity (as it should be in the case of stochastic processes), which confirms the relevance of the results obtained in this case. Conversely, for the samples before incubation or those shielded with foil, the correlation coefficient at t = 0 was approximately 10^–2^. This low value indicates that the correlation function was measured with substantial error, causing the DLS software to mark the results as irrelevant. These findings clearly indicate the absence of a stable well-formed nanobubble phase in the solution prior to incubation or when electromagnetic shielding is applied.

In this regard, let us once again turn to the histograms in Figure 8. The difference between the graphs in panels A and B consists in that the external liquid samples (water) shown in panel B were subjected to vigorous shaking. As shown in our previous work, vigorous shaking of water leads to an increase in the bulk density of gas nanobubbles and excitation of their oscillations (Bunkin et al., 2025). Since these nanobubbles carry an electric charge on their surface, the oscillations of such nanobubbles lead to the generation of electromagnetic radiation in the radio frequency range. The question arises: why does electromagnetic radiation also emerge from the external sample (water), which was not subjected to vigorous shaking (see panel A)? As our previous work showed, the bulk density of ion-stabilized gas nanobubbles increases within water exposed to the atmosphere in a vial for a sufficiently long period of time (Bunkin et al., 2022). This is due to the dissolution of carbon dioxide in the sample, followed by the dissociation of carbonic acid and the formation of bicarbonate ions; these ions act as stabilizers of nanobubbles. Therefore, gas nanobubbles are always present in such water. However, their content is very low, 10^6^–10^7^ cm^−3^ (see above). And seemingly it is this content that predetermines the reference radiation level which can be recorded as shown in Figure 8A. It can be hypothesized that the electromagnetic radiation from water not subjected to vigorous shaking is an evidence of the existence of a physical mechanism for the excitation of oscillations of such nanobubbles. As was demonstrated above, this mechanism is due to the emergence of convective hydrodynamic flows in the water. When the amount of nanobubbles is very low, and the flows, which support their existence, are continuously affected by the outer atmosphere, the oscillations of nanobubbles can scarcely fall in phase. As a result, the radiation level in the outer sample is low and remains nearly unchanged in time. When the flows are predetermined by the temperature gradients and unaffected by the outer atmosphere, as in the case of the closed inner sample, the oscillations of the nanobubbles are less distorted and can fall in phase, which should lead to a slight increase in the recorded radiation (Figure 8A). At the same time, the much larger amount of nanobubbles moving and oscillating in phase due to the vigorous shaking of the outer sample predetermines the much more substantial radiation level and the noticeably larger effect promoted in the inner sample. Insofar as the increase in the radiation level in the inner sample requires the increase in the amount of nanobubbles and their consistent oscillations, this requires a certain time (the aforementioned hour-long delay).

These findings imply that incubation significantly increases the bulk content of gas nanobubbles, providing a qualitative explanation for the results shown in Figure 8.

Furthermore, this model accounts for the enhanced absorption at 600 nm predetermined by the secondary (hydrated) electrons generated upon the high-energy electron beam impact. This phenomenon is attributed to the localization of hydrated electrons within the boundary shells of nanobubbles. As demonstrated in (Novakovskaya et al., 2025), such localization can explain not only the experimentally observed increase in the absorbance at 600 nm (Figure 6, coral markers) but also the slower decay of the spectral signal intensity over time (Figure 7 and the detailed kinetic diagrams in (Novakovskaya et al., 2025)).

We also note that the radiometric setup depicted in Figure 5, containing a portable TES-92 radio frequency radiation detector, remains, in principle, sensitive to the electromagnetic noise effects of the environment. The same can be said about the outer and inner samples in the incubation experiment. And the extent of the corresponding effects cannot be accurately estimated. However, the above interpretation of the experimental results is based on the observed correlations between the recorded radio frequency radiation and the presence of nanobubbles, which, in accordance with our hypothesis, are capable of emitting electromagnetic waves in this range as a result of intense shaking/incubation in an open vessel with the possibility of convective mixing under conditions of developed Rayleigh instability. Apparently, the physical nature of the observed effects remains open to alternative interpretations.

## Conclusion

Our current estimates and proposed explanation of the observed long-range effects are tentative and need further examination and verification. Real liquid phases, such as water or NaCl solutions, contain nanobubbles of different sizes (see the size distributions measured in this study), suggesting that the contributions of diverse nanobubbles (rather than just two kinds, namely, individual nanobubbles of one size and their small aggregates) to the total radiation should be taken into account. Furthermore, the desynchronization of oscillations among these objects should be considered. Independent data regarding the state of the inner and outer solutions adjacent to the flask walls are still required. Nevertheless, the proposed concept represents the first consistent explanation of long-range effects in water based on rigorous physical constructions and model simulations. This model attributes the effects to the presence of charged nanobubbles oscillating in the GHz range (Bunkin et al., 2025). It is nanobubbles that affect the formation and excitation kinetics of hydrated electrons, which appear in water and aqueous solutions upon the high-energy electron beam impact (Novakovskaya et al., 2025). As demonstrated in this study, the synergy of these effects can provide the long-range modification of the properties of an aqueous NaCl solution, which was brought in indirect contact with the vibrationally treated sample, when it is separated from the latter by a liguid non-permeable boundary characterized by a non-zero absorption in the GHz range. We suggest that water and aqueous solutions of salts, which were preliminarily vibrationally treated and then poured into a container open to the atmosphere and experiencing surface evaporation, are capable of emitting electromagnetic waves in the GHz range and, in this way, can affect a sample, which is contained in a tightly sealed flask immersed in this liquid solution open to the atmosphere. Other mechanisms for the long-range interaction of one liquid sample with another may, of course, exist. However, we believe that the suggested mechanism for long-range interaction via the emission of charged nanobubbles oscillating at their eigenfrequency seems quite reasonable. In conclusion, it is worth noting that in natural large water basins (e.g., oceans), where the number of charged nanobubbles of dissolved gas is virtually unlimited and various generation mechanisms of turbulence exist, the existence of stationary electromagnetic noise with a spectral band in the GHz range can be detected. Indeed, such noise emission has been recorded in natural experiments. For example, the polarization properties of noise emission in the GHz range were studied as a function of ocean surface temperature (Shibata, 2006).

## Data Availability

The original contributions presented in this study are included in the manuscript. Further inquiries can be directed to the corresponding authors.
